# The Challenge of Superhydrophobicity: Environmentally Facilitated Cassie–Wenzel Transitions and Structural Design

**DOI:** 10.1002/advs.202305961

**Published:** 2023-12-25

**Authors:** Xin Zhong, Shangzhen Xie, Zhiguang Guo

**Affiliations:** ^1^ Ministry of Education Key Laboratory for the Green Preparation and Application of Functional Materials Hubei University Wuhan 430062 China; ^2^ State Key Laboratory of Solid Lubrication Lanzhou Institute of Chemical Physics Chinese Academy of Sciences Lanzhou 730000 China

**Keywords:** C–W transition, structural design, superhydrophobic

## Abstract

Superhydrophobic materials can be used in various fields to optimize production and life due to their unique surface wetting properties. However, under certain pressure and perturbation conditions, the droplets deposited on superhydrophobic materials are prone to change from Cassie state to Wenzel state, which limits the practical applications of the materials. In recent years, a large number of works have investigated the transition behavior, transition mechanism, and influencing factors of the wetting transition that occurs when a superhydrophobic surface is under a series of external environments. Based on these works, in this paper, the phenomenon and kinetic behavior of the destruction of the Cassie state and the mechanism of the wetting transition are systematically summarized under external conditions that promote the wetting transition on the material surface, including pressure, impact, evaporation, vibration, and electric wetting. In addition, superhydrophobic surface morphology has been shown to directly affect the duration of the Cassie state. Based on the published work the effects of specific morphology on the Cassie state, including structural size, structural shape, and structural level, are summarized in this paper from theoretical analyses and experimental data.

## Graphical abstract

A systematic overview of the behavior and mechanism of C‐W transition of superhydrophobic materials in application environments including pressure, impact, evaporation, vibration, and electrowetting is presented, and the influence of structural design on the stability of Cassie state is also summarized.

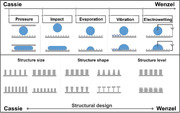

## 1 Introduction

Liquid wetting is an interface phenomenon that occurs in many aspects of life, and this phenomenon has a great influence on many application fields. Inspired by nature, the superhydrophobic phenomenon on the surfaces of lotus leaves,^[^ ^1^ ^]^ roses,^[^ ^2^ ^]^ moth eyes,^[^ ^3^ ^]^ and so on has prompted the discussion of the superwetting properties. The water drops are almost spherical when on the surface of the lotus leaf, and with the slightest disturbance, the water will quickly roll off the surface. This special phenomenon can lead to a wide range of applications, including self‐cleaning,^[^ ^4^ ^]^ anti‐ice,^[^ ^5^ ^]^ antibacterial,^[^ ^6^ ^]^ anti‐corrosion,^[^ ^7^ ^]^ drag reduction,^[^ ^8^ ^]^ and oil/water separation.^[^ ^9^ ^]^ The water droplet on rose's surface is also almost spherical, unlike the phenomenon on the lotus leaf, the water droplet sticks tightly to the surface of the rose, and it does not drop off even when the surface is rotated 180°.^[^ ^10^ ^]^ Slide angle (SA) was introduced to distinguish between phenomena with the same contact angle (CA) but different adhesion properties. In general, a material surface with CA greater than 150° and SA less than 10° is called a superhydrophobic surface. The fabrication of superhydrophobic surfaces depends on the materials with low surface energy and the surface topography. It has been demonstrated that a smooth surface made from material with the lowest surface energy can only reach a CA of 120°, which does not satisfy the definition of a superhydrophobic surface. Therefore, surface morphology is essential for the construction of superhydrophobic surfaces.

A water droplet interacts with a rough surface in three distinct states: complete contact with the rough surface; only partial contact with the top of the rough surface; and the intermediate metastable state. These three different contacts hereinafter are defined as W state, C state, and partial‐C state, respectively. The W state exhibits the largest contact area between the water and a rough surface, followed by the partial‐C state, with the C wetting state having the smallest contact area. The SA value is determined by the pinning action of the three‐phase contact line (TPCL), and it is influenced by the adhesion force of water interacting with the rough surface.^[^ ^11^ ^]^ The adhesion force between water and rough surface is at its maximum in the W state and at its minimum in the C state, originating from the action force which increases with the increase in the contact length. In simpler terms, the C state has a smaller SA and can more easily remove water droplets from the material surface. When comparing the W and C states, the main difference lies in the gas contact area. The gas that exists between the surface morphology and the water is referred to as an air pocket (plastron). The contact area fraction and volume of gas pockets influence the value of CA and the duration of the C state, respectively. These factors ultimately determine the lifespan of superhydrophobic materials. However, current research suggests that the superhydrophobicity of the C state is quite fragile. The rough structure of the material surface may lose its superhydrophobicity after experiencing wear. Moreover, even if the material surface remains undamaged, it may lose its C state and transition to the W state, which has a higher SA value, under certain conditions such as pressure, droplet impact, evaporation, vibration, and electrical wetting. For processes like coating, printing, and painting, maintaining the W state is beneficial. However, for superhydrophobic applications, a stable C state is necessary.^[^ ^12^ ^]^ Numerous researchers have constructed various forms of C surfaces and conducted extensive experiments and theoretical analyses to explore the mechanism and mode of the Cassie–Wenzel transition (C‐W transition).

Understanding the C‐W transition mechanism is of great significance for prolonging the service life of superhydrophobic surfaces. In this paper, based on the investigation of current research on the stability of the C state, the description and the analysis methods of C state stability are summarized, the C‐W transition mechanism is discussed, and the influence of the application environment and the material surface morphology on C state stability is analyzed. This review offers guidance on the fabrication of superhydrophobic surfaces.

## 2 Fundamental Understanding of Superhydrophobicity

### 2.1 Superhydrobicity and Wetting States

#### 2.1.1 Young's Equation

When sessile droplets rest on a solid surface, they can take on different equilibrium states. Oil droplets tend to spread out flat, while water droplets form a shape like a ball‐cap. This is due to differences in surface tension, which leads to different CA values. In 1805, Thomas Young introduced an equation that describes the balance between surface tension and CA on a completely homogeneous and smooth horizontal surface:^[^ ^13^ ^]^

(1) $$\gamma_{\mathrm{LG}}\cos\;\theta_{Y}=\gamma_{\mathrm{SG}}\;-\gamma_{\mathrm{SL}}$$

Where *γ*_SL_ is the solid–liquid interfacial tension, *γ*_SG_ is the solid–gas interfacial tension, and *γ*_LG_ is the liquid–gas interfacial tension. *θ*_Y_ is the angle between the tangent line of the water drop surface and the horizontal plane. *θ*_Y_ is the value of the CA when the chemical composition is uniform and the surface is smooth, also called the intrinsic CA. According to the value of the CA, the liquid–solid wetting forms can be simply categorized into two types with 90° as the dividing line: CA < 90°, hydrophilic state; CA > 90°, hydrophobic state. According to Young's equation, the CA value depends solely on the surface tension values of the three phases. However, in real‐world scenarios, the value of the CA differs from the value calculated in Young's equation due to the influence of some factors, including the microscopic heterogeneity of the solid surface, the pH of the liquid,^[^ ^14^ ^]^ and the volume of the liquid.^[^ ^15^ ^]^ The volume of the droplet greatly affects the measured CA. Larger droplets increase the effect of gravity, leading to a larger deviation.^[^ ^16^ ^]^ To minimize gravity's influence, the measured droplet is generally smaller than the characteristic length ($l\;={\left(\frac{\gamma }{\rho g}\right)}^{\frac{1}{2}}\;$, where *γ* is the surface tension, *ρ* is the liquid density, and *g* is the gravitational acceleration).^[^ ^17^ ^]^ For water droplets, the characteristic length is 2.7 mm. When measuring a material's CA with water droplets, the diameter of the water droplets should be controlled to be less than 2.7 mm. Medale et al.^[^ ^18^ ^]^ suggested that under weightless conditions, the volume range for hydrostatic equilibrium CA is much larger than under gravity conditions. Its value gradually converges to that measured by Young's equation in an approximately positive limit, providing a new perspective for applying Young's equation.

#### 2.1.2 Wenzel's Equation

Due to the completely uniform and smooth surface does not exist in reality. Wenzel introduced the concept of roughness (*r*) based on Young's equation to better represent the real surface.

(2) $$r\;=\frac{A_{\mathrm{actual}}}{A_{\mathrm{projection}}}\;$$

Where, *A*_actual_ refers to the actual total area of the material surface, and *A*_projection_ refers to the projected area. Wenzel's equation is given to calculate the CA on the rough surface:^[^ ^19^ ^]^

(3) $$\cos\;\theta_{w}=\;r\cos\theta_{Y}$$

Where, *θ*_w_ is the CA under the W state, which is when the droplet is in full contact with the rough surface. Researchers refer to the fully wetted state as the W state. Since material surfaces are always rough, *r* is always greater than 1, which amplifies the wettability of the material. In other words, if a material is inherently hydrophilic, increasing its roughness makes it more hydrophilic; if a material is inherently hydrophobic, increasing its roughness makes it more hydrophobic. This amplification is not linear. When the CA is close to 90°, the amplification effect is significant. But when the CA is close to 0° or 180°, the amplification effect becomes less pronounced.

#### 2.1.3 Cassie–Baxter Equation

In reality, Wenzel's equation does not fully explain many superhydrophobic phenomena found in nature, such as the low adhesion of lotus leaf surfaces. If the liquid were in complete contact with the surface of the lotus leaf, there is only the liquid–solid interface beneath the entire liquid. The roughness of the leaf would increase its area of action, and the corresponding total adhesion force should also increase. However, this contradicts the observed low adhesion of the lotus leaf. To address this, Cassie introduced the concept of an air pocket.^[^ ^20^ ^]^ When a liquid is deposited on a material's surface, it does not completely contact the solid surface. There is some gas trapped between the rough structures.^[^ ^21^ ^]^ Therefore, the actual contact interface should be considered a composite of a gas–liquid interface and a solid–liquid interface. This led to the proposal of the Cassie–Baxter equation:

(4) $$\cos\;\theta_{c}=f_{1}\;\cos\theta_{1}+f_{2}\cos\theta_{2}$$

Where, *f* represents the contact area fraction of a single component in the composite interface, with different components distinguished by subscripts. *θ*_1_ is the intrinsic CA of *f*_1_. Similarly, *θ*_2_ is the intrinsic CA of *f*_2_. For a composite interface of water and solid‐gas, one group is considered a gas–liquid interface. Let *f*_2_ represent the liquid–gas interface area fraction, and then *f*_1_ represents the liquid–solid interface area fraction. The sum of *f*_1_ and *f*_2_ equals 1. So *f*_2_ can also be expressed as:

(5) $$f_{2}=\;1-f_{1}$$

At this point, *θ*_2_ = 180°. So the Cassie–Baxter equation can also be expressed as:

(6) $$\cos\;\theta_{c}=f_{1}\;\left(\cos\theta_{1}+1\right)-1$$

This equation simplifies calculations for models like the wetting state of a lotus leaf surface, commonly referred to by researchers as the C state. In other words, water does not completely wet the material surface upon contact, and there is air in between. The wetting interface is a composite wetting interface of water and solid–gas.

#### 2.1.4 Double Layer Surface Wetting Equation

**Figure 1:**
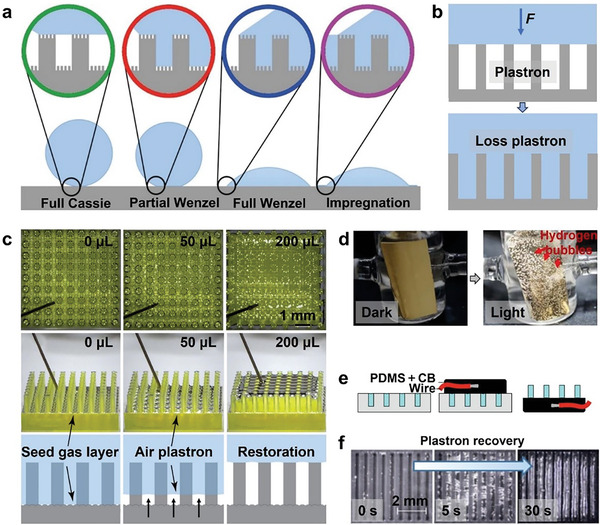
a) Wetting states on a two‐tier surface. Reproduced with permission.^[^ ^22^ ^]^ Copyright 2011, American Chemical Society. b) Schematic diagram of the wetting transition of the system after the loss of plastron. c) The top view (top), side view (middle), and process diagram (bottom) of the successful plastron recovery experiment through air injection. Reproduced with permission.^[^ ^39b^ ^]^ Copyright 2022, American Chemical Society. d) Digital images demonstrating the regeneration of underwater superhydrophobicity through the generation of hydrogen gas bubbles in the dark and light. Reproduced under terms of the CC‐BY license.^[^ ^40^ ^]^ Copyright 2015, The Authors, Published by Springer Nature. e) Fabricate the surface with a molding process: a mixture of PDMS and carbon black (CB) is covered with embedded wire on top of the mold and left to cure. Then the surface is demolded. f) As the circuit is closed, full plastron recovery from a fully wetted state. Reproduced under terms of the CC‐BY license.^[^ ^41b^ ^]^ Copyright 2022, The Authors, Published by American Chemical Society.

The Cassie–Baxter equation aligns well with the wetting phenomenon of a single‐scale composite interface. However, for micro–nano interfaces, there are various impregnation states due to the different wetting difficulties of micron and nanoscales. Therefore, the Cassie–Baxter equation needs some modifications. Wetting can be divided into four cases based on the degree of wetting: full C state, partial W state, full W state, and impregnation state (**Figure** **1**a).^[^ ^22^ ^]^ For the CA model in full C state, the equation is modified to:

(7) $$\cos\;\theta_{\mathrm{fc}}=f_{m}\;f_{n}\left(\cos\theta_{Y}+1\right)-1$$

Where, *f*_m_ and *f*_n_ are solid parts with micron and nanoroughness, respectively. The equation of the partial W state for wetting only the micron structure is modified as follows:

(8) $$\cos\;\theta_{\mathrm{pw}}=\left(r_{m}+f_{n}-1\right)\;\cos\theta_{Y}+f_{n}-1$$

Where, *r*_m_ is the roughness of the micron scale. The CA equation for the full W state where both micron and nanoscales are wetted is:

(9) $$\cos\;\theta_{\mathrm{fw}}=\left(r_{m}+r_{n}-1\right)\;\cos\theta_{Y}$$

Where, *r*_n_ is the roughness of the nanoscale. The impregnation state not only wets the micron and nanoscale structures but also extends to the surrounding liquid film formation. The CA equation is:

(10) $$\cos\;\theta_{i}=f_{m}\;f_{n}\left(\cos\theta_{Y}-1\right)+1$$

### 2.2 Some Factors for Assessing C State Stability

#### 2.2.1 Contact Angle

When a water droplet is in equilibrium on the surface of a material, the tangent line of the side in contact with the gas and the solid–liquid contact plane form an angle, which is defined as the CA. The CA is used to describe the wetting state of the liquid on the material's surface. The value ranges from 0° to 180°. The size of the CA reflects the droplet's interaction with the material surface, which is related to the surface tension.^[^ ^23^ ^]^ When the surface tension value of the material is constant, the smaller the surface tension of the droplet, the easier it is for the droplet to wet the material surface, which is reflected in a smaller CA. Vice versa, if the droplet's surface tension is higher, it will be more difficult for the droplet to wet the material surface, resulting in a larger CA. It follows the principle of overall minimum energy. The volume and temperature of the droplet influence the CA value.^[^ ^24^ ^]^ Contamination or ionic interference in the substrate can significantly affect CA.^[^ ^25^ ^]^ The substrate's curvature also plays a role in determining the CA value, with a decrease on concave substrates and an increase on convex substrates. This effect of curvature is more pronounced on substrates with higher wettability.^[^ ^26^ ^]^

#### 2.2.2 Contact Angle Hysteresis

Due to the fact that surfaces are not perfectly smooth, TPCL pins suffers from surface deformation, roughness, and non‐homogeneity, leading to hysteresis in the fluid's movement across the surface.^[^ ^27^ ^]^ Simply employing CA to describe the wetting phenomenon of liquid cannot reflect the dynamic behavior of liquid. The critical CA values for the expansion and retraction of water droplets in the horizontal plane after injection and extraction are defined as the advancing angle (*θ*_adv_) and receding angle (*θ*_rec_), respectively. For inclined plates, *θ*_adv_ and *θ*_rec_ are two angles prior and subsequent to when the droplet is about to roll. Contact angle hysteresis (CAH) is defined as the difference between the *θ*_adv_ and the *θ*_rec_ to describe a dynamic wetting behavior.^[^ ^28^ ^]^ The CAH is caused by the force between the TPCL and the substrate, which operates at the scale of microns, nanometers, and atoms.^[^ ^29^ ^]^ At the microscopic scale, the local CA varies due to the varying interaction forces between the contact line and the local substrate.^27^, ^30^ ^]^ Therefore, the value of the CAH can reflect the adhesion of the liquid to the substrate.^[^ ^31^ ^]^ According to the Young‐Dupré formula:^[^ ^32^ ^]^

(11) $$W_{\mathrm{ad}}=\gamma_{\mathrm{LG}}\;\left(1+\cos\theta_{\mathrm{adv}}\right)$$

where, *W*_ad_ is the adhesion force between the liquid and the substrate. The CAH of the droplet is greater in the W state and smaller in the C state.

#### 2.2.3 Plastron's Volume

The existence of an air pocket/plastron between the C‐state substrate and the liquid is what sustains its application,^[^ ^33^ ^]^ including self‐cleaning and drag reduction.^[^ ^34^ ^]^ These applications are typically immersion‐based. The core issue is the resistance and durability of the plastron under pressure.^[^ ^35^ ^]^ Under specific external circumstances, the gas could be displaced, or expelled by the liquid, or diffused into the liquid, leading to a failure of superhydrophobicity (Figure 1b). This could be due to factors including high hydraulic pressure, droplet impact, surface vibration, and the presence of an electric field. As air diffuses into the liquid, several factors, including surface tension, pressure, temperature, and concentration of dissolved gas, influence the formation and persistence of cavitation by managing the rate of gas diffusion.^[^ ^36^ ^]^ Furthermore, when the surface is subjected to a flowing liquid, the shear flow and its associated stress gradient contribute to a decrease in cavitation air.^[^ ^37^ ^]^ Positive and negative pressure differences can lead to cavitation or nucleation.^[^ ^38^ ^]^ Once cavitation is depleted, the entire system enters a W state, leading to a loss in application performance. Cavitation can be recovered through active strategies, including air injection,^[^ ^37^, ^39^ ^]^ photocatalysis,^[^ ^40^ ^]^ and electrolysis (Figure 1c–f).^[^ ^41^ ^]^ The passive strategy influences the presence time of cavitation by controlling the substrate surface morphology (e.g., *Salvinia* leaf can maintain a longer C state underwater due to its unique egg‐beater structure).^[^ ^35^, ^42^ ^]^ The cavitation time can be prolonged by managing the surface characteristics of the substrate, including the spacing and height between the microstructures, as well as the Springtails’ reentrant structures and micro‐nanohierarchical structure.

### 2.3 Meniscus Stability Analyses

#### 2.3.1 Thermodynamic Free Energy Analyses

Thermodynamic analysis is a method used to study the energy changes and equilibrium states of a substance or system. Thermodynamic analysis can be used to assess the possibility and stability of the wetting transition, as well as the efficiency and optimization of the wetting process. A large number of thermodynamic studies have been conducted to analyze the C‐W transformation mechanism. This ranges from analyzing simple 2D models^[^ ^43^ ^]^ to more complex three‐dimensional models,^[^ ^44^ ^]^ along with simulations and assessments of single, uniformly distributed arrays and complex, diverse microstructurally arranged surfaces. Moreover, it extends from general thermodynamic analyses to the exploration of specific model simulations. Overall, these studies provide a significant reference for the development of practical microstructures. The thermodynamic simulation technique is employed to create models that consider perspectives ranging from macroscopic to microscopic and mesoscopic, encompassing molecular dynamics simulation,^[^ ^45^ ^]^ finite element analysis,^[^ ^46^ ^]^ atomic simulation,^[^ ^47^ ^]^ microscopic density functional analysis,^[^ ^48^ ^]^ cellular Potts model,^[^ ^43^, ^49^ ^]^ and lattice Boltzmann method.^[^ ^50^ ^]^ Investigating the energy transformation and energy barriers of the system offers direction for the design of surface morphology.

The most commonly used method for thermodynamic simulation of the wetting transition is finite element analysis.^[^ ^44^, ^51^ ^]^ The entire composite system is broken down into a multitude of small, simple units that interact with one another, and the small units are analyzed energetically. The motion environment is simplified, and the energy of all the small units is integrated to deduce the energy variation of the whole system.

For the energy analysis of the W state and C state, the W state has a relatively low free energy state, while the C state has a relatively high free energy state.^[^ ^44a^ ^]^ This indicates that the W state is more stable from a free energy minimization perspective, while the C state is thermodynamically unstable. However, in the actual experiment, the C state will not suddenly transform into the W state without any external influence. The C state contains air pockets that inhibit the droplets from moving forward and causing wetting. This is demonstrated in the free energy curve as an increase in the free energy value, that is, the whole system needs to overcome a specific energy barrier during the transition process. However, this barrier can be easily overcome with external intervention to achieve the C‐W transition. From a thermodynamic standpoint, reducing the free energy difference between the C state and W state and elevating the energy barrier of the transition contribute to maintaining the stability of the C state.

#### 2.3.2 Mechanical Analyses

In addition to the thermodynamic free energy analysis of the lowest energy state, equilibrium force analysis of the system by mechanical methods can also elucidate the reasons behind the wetting transition. By examining the mechanical composition of the entire system and the dynamic behavior of the fluid during the wetting process, the characteristics and rules of interface deformation can be investigated. The fundamental process behind the wetting transition is the liquid‐air interface collapsing due to the disruption of equilibrium within the liquid‐gas‐solid TPCL beneath water.^[^ ^38^, ^52^ ^]^ In terms of mechanics, the wetting transition occurs when the downward driving force exceeds the upward resistance.^[^ ^53^ ^]^ In the absence of external fields, the mechanical analysis is relatively simple. When external fields are introduced, local complex force distribution calculations are required for the entire system, including the magnetic field and the electric field.^[^ ^52a^ ^]^ Annavarapu et al.^[^ ^52b^ ^]^ introduced the concept of critical force value per unit length to explain the wetting transition mechanism from the local force balance model.

Mechanical analysis requires careful consideration and a specific analysis of the environment in which the liquid is located. Liquids exert different forces in different ways. For submerged liquid surfaces, the stability of the wetting regime depends on the hydrostatic pressure.^[^ ^36^, ^54^ ^]^ When a liquid hits a surface at a certain velocity, the water hammer pressure is the main driving force.^[^ ^55^ ^]^ The internal flow caused by evaporation enhances the internal pressure of the droplet and drives the wetting transition.^[^ ^56^ ^]^ Similarly, mechanical analysis can be utilized to assess the resistance of different structural models to applied forces, which is instructive for the design of surfaces with stable C states.^[^ ^57^ ^]^

### 2.4 Two Models of the Cassie–Wenzel Transition

**Figure 2:**
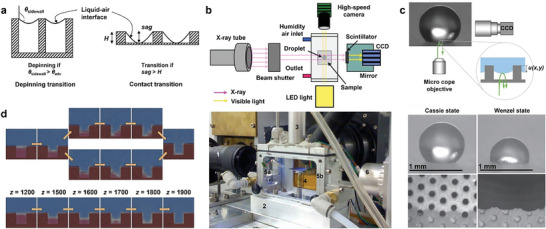
a) Side views of the C‐W transition due to depinning and sag mechanisms. Reproduced with permission.^[^ ^59a^ ^]^ Copyright 2010, American Chemical Society. b) Schematic of X‐ray photography captures observation means (top) and picture of (1) the optical lens, (2) the environmental chamber, (3) the falling drop guide tube, (4) the sample on the translation and tilting stage, (5a,b) the relative humidity sensors, and (6) the LED illuminator (bottom). Reproduced under terms of the CC‐BY license.^[^ ^53a^ ^]^ Copyright 2014, The Authors, Published by Springer Nature. c) Schematic representation of the RICM visualization of air observation means (top), and the side view and RICM photo of the water drop to the PDMS in C state (left) and W state (right) respectively (bottom). *u*(*x*,*y*) is the liquid–air interface profile. Reproduced with permission.^[^ ^60^ ^]^ Copyright 2007, EDP Sciences, Società Italiana di Fisica and Springer‐Verlag. d) Two wetting paths of smooth instantaneous density field (top) and average density field (bottom). Z stands for direction. Reproduced with permission.^[^ ^61^ ^]^ Copyright 2012, American Chemical Society.

Understanding the mechanism of the C‐W transition of droplets on material surfaces is crucial, as it holds significant value in determining the C‐W transition and provides theoretical support for the development of a stable C surface. One of the criteria for assessing the C‐W transition is whether the droplets are in contact with the bottom of the rough groove.^[^ ^58^ ^]^ The researchers, after a number of transition experiments and theoretical analysis, identified two types of C‐W transition patterns: the depinning transition and the contact transition (**Figure** **2**a).^[^ ^59^ ^]^ The trend of the subsurface interface can be observed by observational means such as X‐ray photographic capture (Figure 2b)^[^ ^53a^ ^]^ and reflection interference contrast microscopy (RICM) visualization of air (Figure 2c).^[^ ^60^ ^]^ When the droplet is on the surface of the material through a certain action mode, the solid‐liquid interface forms a certain angle with the contact part of the microstructure edge (i.e., groove/column sidewall angle) on the rough surface. The applied external force induces an increase in the curvature of the interface under the liquid, and the groove/column sidewall angle increases. When the groove/column sidewall angle exceeds the *θ*_adv_, the liquid surface is de‐pinned at the edge of the groove/column. The contact line of the liquid level will move down continuously along the sidewall of the groove column until the bottom of the meniscus touches the substrate, triggering an instantaneous wetting transition. This transition mode is called the depinning transition. The contact transition means that the lowest part of the concave surface under the liquid is already in contact with the substrate when the angle of the groove/column sidewall increases with the curvature of the interface under the liquid but does not reach *θ*_adv_, and then the substrate is quickly wetted. It is generally recognized that the interface shows a symmetric curvature of the meniscus during the wetting transition. Giacomello et al.^[^ ^61^ ^]^ analyzed the wetting of the nanogroove surface by atomic simulations based on energy minimization and found that the C‐W transition followed an asymmetric path with two forms in instantaneous density field, that is, liquid on one side of the groove and bubble on the other side. The average of all paths resulted in a conventional parallel meniscus path (Figure 2d). It is explained by a macroscopic argument based on the minimization of the free energy of the surface. When the groove is filled with only a small amount of liquid, the liquid with a flat curved meniscus has the smallest free energy, while when enough liquid penetrates the groove, the curved meniscus confined between the bottom and the side walls of the groove has a smaller liquid vapor area and therefore has a lower free energy than the flat curved meniscus.^[^ ^62^ ^]^ This results in an asymmetric path when wetting transitions occur.

## 3 Environmental Factors Affecting the Stability of C State

The instability of the C state was discovered when researchers explored superhydrophobic surfaces. The fragile, rough structure on the superhydrophobic surface quickly loses its superhydrophobicity after undergoing wear. Maintaining the long‐term wear resistance of superhydrophobic surfaces is a difficult problem to overcome. Wang et al.^[^ ^63^ ^]^ constructed a micro–nano composite structure that provides highly hydrophobic nanostructures by using the frame microstructure as an “armour” to effectively improve the wear resistance of the material. Nonetheless, under specific environmental conditions, including pressure, impact, evaporation, vibration heating,^[^ ^64^ ^]^ electric field,^[^ ^52a^ ^]^ and gravity,^[^ ^65^ ^]^ the superhydrophobic surface may lose its superhydrophobicity due to the C‐W transition, even if the surface appears undamaged. This chapter discusses only the influence of the environment on the C state stability and assumes that the surface morphology and structure of the material are not damaged, or only slightly damaged, and the effect on the surface droplet state can be neglected.

### 3.1 Pressure

**Figure 3:**
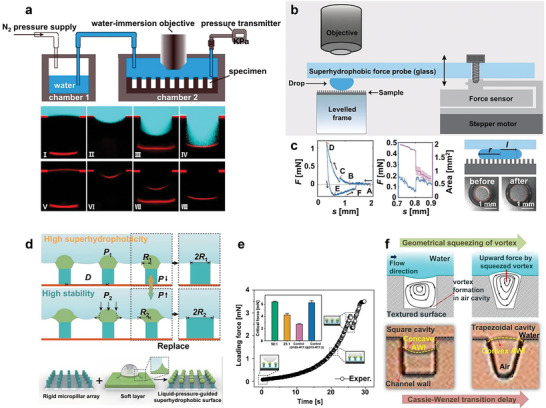
a) Schematics of the hydraulic test device experiment (top), and confocal microscopy images (bottom) showing the meniscus 5 min after fresh immersion under 0 kPa (I),(V), 14 kPa (II),(VI), 50 kPa (III),(VII), and 15 min after immersion under 50 kPa (IV),(VIII). Images in the first row were acquired with fluorescence‐labeled water; the second row images were acquired without the dye. Reproduced with permission.^[^ ^68^ ^]^ Copyright 2014, American Physical Society. b) Sketch of the extrusion experimental device setup. The force probe (superhydrophobic glass slide and force sensor) is moved by the vertical translation stage. c) Typical force‐separation curve of a squeezed drop between the two superhydrophobic surfaces: force probe and micropillar array. The arrows represent the progression followed during the experiment, starting at A and finishing at F (left). Images before and after C‐W transition, the edges of the contact lines are marked with red lines (right). Reproduced with permission.^[^ ^69a^ ^]^ Copyright 2022, American Chemical Society. d) Schematic showing the principle of the adaptive radius of the soft head in response to the infiltration pressure (*R*_2_ > *R*_1_, *P*_2_ > *P*_1_) (top). Self‐adaptive superhydrophobic surface manufactured from rigid micropillars with a soft head (bottom). e) Critical force exerted on the water droplet when impalement happened on the liquid‐pressure‐guided superhydrophobic surface with different rigidities of the hemisphere head. 50:1, 25:1 represents the ratio of PDMS crosslinkers, and 50:1 is more like elastic materials. The control surface (D100‐R17.5) (D75‐R17.5) with a cross‐linker ratio of 10:1 is shown as a rigid surface. Reproduced with permission.^[^ ^70^ ^]^ Copyright 2022, Wiley‐VCH GmbH. f) Dynamics and visualization of liquid–gas interface of square, and trapezoidal cavities under flow conditions. The air–water interface (AWI) is marked by a yellow dotted line. Reproduced with permission.^[^ ^54^ ^]^ Copyright 2015, American Chemical Society.

The superhydrophobic surface will inevitably experience a certain level of pressure in the process of application. This could either be due to a single droplet being squeezed or the hydraulic pressure of the entire liquid surface being applied. For instance, a certain separation pressure is required for the oil/water separation.^[^ ^66^ ^]^ Pressure is very common for the C state to break down.^[^ ^67^ ^]^ Based on practical observations, researchers have found that the cavity volume of a superhydrophobic surface decreases under pressure until a C‐W transition occurs. The mechanism of pressure influence on the wettability transition needs to be explored. C‐state stability is usually evaluated and contrasted with the critical pressure (the pressure at which the droplet begins to occur during the C‐W transition) and the critical time (the time from applying the pressure to the completion of the C‐W transition, that is, the time of cavity collapse). The test of critical pressure and time is divided into two modes: a) Impregnate the material under the water column of a certain height and calculate the pressure value by (**Figure** **3**a):^[^ ^68^ ^]^

(12) P=ρgh

Where, *h* is the height of the liquid. The C‐W transition can be quantified by observing the luminance of the gas cavity through light scattering or laser confocal microscopy. The latter technique allows for visualizing the position of the TPCL and the volume of the air cavity during the C‐W transition.^[^ ^68^ ^]^ b) A drop of water is placed on the sample surface, with a plate parallel to the horizontal direction positioned above the drop (Figure 3b). The motor drive is used to control the movement of the plate.^[^ ^69^ ^]^ A force sensor is used to reflect the pressure values. Pressure values at different times were recorded and plotted. The sudden spikes in the pressure‐time graph can directly reflect the critical pressure during the C‐W transition (Figure 3c). The meniscus is observed in both experimental models at fluid pressure. Local hydraulic pressure above the cavity drives the fluid–gas interface into the cavity, while surface tension at the interface and air pressure in the cavity resist fluid invasion.^[^ ^54^ ^]^ As pressure mounts, the curvature of the meniscus continues to rise, leading to the depinning transition and the contact transition. The *θ*_adv_ is the critical value of these transitions, and the roughness of the sidewall influences the *θ*_adv_ value.^[^ ^69b^ ^]^ The mode of transition can be controlled by the height of the microcolumn. The rough surface morphology of the material impacts the critical pressure (see Section 4.2). The pressure applied from the exterior influences the duration of the critical state. An increase in pressure leads to a shortening of the critical transition time.^[^ ^68^ ^]^ In order to achieve the realistic requirement for a continuous C state, the C state should be extended by minimizing the external forces or changing the structural factors of the material surface. Tan et al.^[^ ^70^ ^]^ published that by designing the structure of the material into a soft and hard combination mode, hydraulic pressure was used to change the shape of the soft head, so as to realize the long diameter and low spacing surface structure under the condition of high hydraulic pressure, so as to extend the C state of the material surface (Figure 3d,e).

The aforementioned only considers hydrostatic pressure. In addition, the flow application environment also needs to be considered. Dynamic superhydrophobic stability is crucial for the application of superhydrophobic surfaces. The velocity of fluid flow affects the entire wetting system. As the flow rate escalates, the local pressure difference at the liquid–gas interface overcomes the capillary pressure of the cavity. As a result, the liquid–gas meniscus at the edge of the cavity is removed until the C‐W transition occurs. The fluid flow causes eddy motion in the cavity, and the resulting force can hinder the C‐W transition at the interface. For cavities of different shapes, inclined‐wall trapezoidal cavity exhibit a delayed C‐W transition compared with square cavities (Figure 3f).^[^ ^54^ ^]^ At the same time, the gas diffusion affected by the difference in air content in water also affects the C‐W transition.

### 3.2 Impact

When water droplets hit the material surface at a certain speed, the impact kinetic energy may trigger the C‐W transition. A range of facilities and equipment that need to work in the outside environment, like aircraft, automobiles, and power stations, can be subjected to intense impacts on rainy days, resulting in superhydrophobic surfaces that are challenging to effectively utilize. For dispensing technology where tiny droplets act on the surface of the material, such as inkjet printing, the droplet impact behavior greatly affects the processing accuracy.^[^ ^71^ ^]^ In recent years, numerous researchers have conducted extensive research on the wetting dynamics, wetting transition, and different application environments of droplet impact to liquid surface from theoretical simulation and experimental operation.^[^ ^72^ ^]^

**Figure 4:**
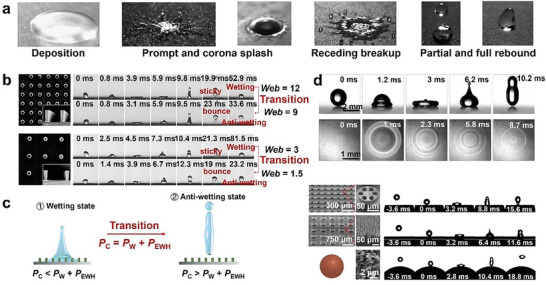
a) Outcomes of drop impact: deposition, prompt splash, corona splash, receding breakup, partial and full rebound. Reproduced with permission.^[^ ^73^ ^]^ Copyright 2015, Elsevier B.V. b) Snapshots of the impact dynamics of water droplets showing the transition between wetting/anti‐wetting states at different *Web* number. c) Mechanical analysis diagram describing the transition from wetting state to anti‐wetting state. Reproduced with permission.^[^ ^55^ ^]^ Copyright 2017, The Royal Society of Chemistry. d) The droplet complete rebound image when *Web* = 10 and the interference fringe image of RICM imaging (top). Snapshots showing the droplets impacting on oil‐infused mushroom structure (easy infiltration of droplets leads to more viscous dissipation), pyramid arrays, and spherical surface (droplets do not penetrate easily; bottom). Reproduced under terms of the CC‐BY license.^[^ ^80^ ^]^ Copyright 2015, The Authors, Published by Springer Nature.

When a droplet hits a solid surface from a certain height, it will be in six states after interacting with the surface: deposition, partial rebound, full rebound, prompt splash, corona splash, and receding breakup (droplet fragmentation and satellite droplet generation) (**Figure** **4**a).^[^ ^73^ ^]^ The manner in which a droplet deforms upon collision with a superhydrophobic surface mainly depends on its impact velocity, or kinetic energy of impact. As the impact velocity increases, the droplet appears to bounce, deposit, and break. The ultimate form of the bouncing droplet on the material surface is the C state. The deposited and broken droplets are in the W state. The dynamics of droplet impact can be described by various dimensionless numbers, including the *Weber* number ($Web\;=\frac{\rho D_{0}U_{0}^{2}}{\sigma }$), the *Reynolds* number ($Re\;=\frac{\rho D_{0}U_{0}}{\mu }$), the *capillary* number ($Ca\;=\frac{We}{Re}$), and the *Ohnesorge* number ($\mathrm{Oh}\;=\frac{\mu }{\sqrt{\rho \sigma D_{0}}}$), where *U*_0_ is the impact velocity, *D*_0_ is the diameter, and *µ* is the viscosity.^[^ ^74^ ^]^ In general, researchers use the *Web* as a direct substitute for impact velocity. Given that the examined droplets are water, the density, characteristic length, and surface tension coefficient remain constant, with only the impact velocity of the droplets varying. *Web* values can directly reflect the impact of kinetic energy and the effect of surface tension on the wetting transition. When the value of *Web* is under 1, it indicates that surface tension plays a major role, and when the value of *Web* is over 1, it indicates that impact is the key cause of the wettability transition. Typically, researchers ensure that the *Web* number is greater than 1 when examining the effect on the wetting transition. The impact kinetic energy is positively correlated with the impact velocity and the *Web* number. As the *Web* number increases, the droplet tends to deform, diffuse, and wet the surface, potentially even breaking apart (Figure 4b).^[^ ^55^, ^73^, ^75^ ^]^

From a mechanical point of view, when a droplet hits a surface, it generates dynamic pressure ($P_{W}=\;\rho U_{0}^{2}/2$), hammer pressure ( *P*_EWH_ =  *kZ*_e_ *V*, where *k* is an empirical constant, *Z*_e_ is the effective acoustic impedance of the liquid, and *V* is the volume. The effective acoustic impedance is a combination of liquid and substrate acoustic impedances, *Z*_l_ and *Z*_s_, respectively. Acoustic impedance, which for any material is a product of its density and speed of sound through it, and capillary pressure.^[^ ^55^, ^76^ ^]^ Water hammer pressure and dynamic pressure generally promote droplet penetration into micro/nanostructure cavitation, whereas capillary pressure inhibits it. When the capillary force surpasses both the dynamic pressure and the water hammer pressure, the droplet will bounce off the surface completely (Figure 4c). Long aspect ratio structure and tip structure can enhance capillary force.^[^ ^77^ ^]^ From the perspective of thermodynamics, as the droplet hits the surface, part of its initial kinetic energy transforms into surface energy due to the increased diffusion area. The kinetic energy of a droplet can be defined as the volume integral of the kinetic energy of an infinitesimal volume element *V*_0_ in a liquid medium:

(13) $$K\;=\int \frac{1}{2}\;\rho U_{0}^{2}dV_{0}$$

And surface energy:

(14) $$S\;=\gamma_{\mathrm{LG}}\;S_{a}+\left(\gamma_{\mathrm{SL}}-\gamma_{\mathrm{SG}}\right)S_{s}$$

Where *S*_a_ and *S*_s_ are the regions where droplets come into contact with gas and solid medium, respectively.^[^ ^78^ ^]^ Part of the kinetic energy is converted to viscous dissipation due to viscous dissipation within the liquid medium or liquid–solid interaction (friction and TPCL pinning).^[^ ^78^, ^79^ ^]^ As the droplet reaches its maximum diffusion radius, the stored surface energy begins to convert into kinetic energy, causing the droplet to shrink toward the impact point. The determining factor of the impact effect is the energy dissipation during diffusion. The pinning of contact lines due to droplet infiltration is the main source of viscous dissipation. Reducing the energy dissipation caused by contact line pinning, thereby allowing more energy to be used to promote partial rebound of the surface during the retraction phase (Figure 4d).^[^ ^78^, ^79^, ^80^ ^]^ The droplet can be separated from the surface at the end of the retraction phase if the droplet is converted to rebound kinetic energy high enough after overcoming the energy consumed by the pin dissipation of the TPCL and the viscous dissipation within the liquid medium. Subsequently, the droplet is in the process of converting kinetic energy to surface energy and accompanied by viscous dissipation, exhibits bouncing behavior. When the droplet stops moving, the viscous dissipation disappears. Increasing the hydrophobicity of the solid surface can reduce the energy consumed during impact.

The duration of contact between the droplet and the surface influences the transfer of mass, momentum, and energy.^[^ ^81^ ^]^ Impact contact time is divided into diffusion time and retraction time.^[^ ^82^ ^]^ Richard et al.^[^ ^83^ ^]^ discovered that the droplet contact time is a function of droplet mass and liquid surface tension and is unrelated to the impact velocity:

(15) $$t_{R}=\;2.6{\left(\frac{\rho D_{0}^{3}}{8\sigma }\right)}^{\frac{1}{2}}$$

Reducing the contact time and boosting the conversion of surface energy to kinetic energy enables the droplet to rebound, that is, the C state. Splitting impact droplets into smaller pieces is an effective method to shorten the contact time on the superhydrophobic surface.^[^ ^84^ ^]^ Huang et al.^[^ ^77^ ^]^ demonstrated that after a droplet hits the surface edge and breaks apart, the inertial mass of the main droplet decreases and its retraction momentum increases, thus reducing the contact time of the impacting droplet. Liu et al.^[^ ^85^ ^]^ observed the pancake shape of the liquid on impact on the conical column because sufficient energy storage allows the liquid to quickly break away from the surface, and its contact time is fourfold shorter than that of traditional rebound. Tang et al.^[^ ^86^ ^]^ also observed the elastic enhancement behavior of the conical surface, proving that it is achieved by sacrificing the flow redirection effect on the conical surface structure, thus reducing the viscous dissipation and increasing the rebound energy.

**Figure 5:**
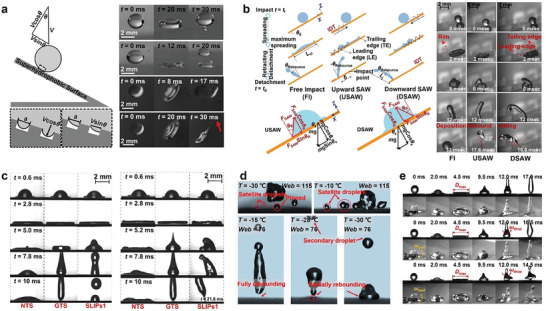
a) Schematic of a drop impact and the zoomed in view of the liquid–air interface: Decomposition of velocity into normal and tangential components (left) and the images of plane and slope behavior at different altitudes and different speeds. From top to bottom are: Complete rebound on a horizontal, post‐textured surface, and release height *H* = 0.02 m, *V* = 0.62 m s^−1^. Partial rebound on the same surface, *H* = 0.03 m, *V* = 0.77 m s^−1^. Drop impact on a 60° slanted surface with complete rebound, and release height *H* = 0.10 m, *V*cos*θ* = 0.70 m s^−1^. Drop impact on the same surface with partial rebound and *H* = 0.13 m, *V*cos*θ* = 0.8 m s^−1^. Reproduced with permission.^[^ ^87^ ^]^ Copyright 2015, Elsevier Inc. b) Schematic views of different scenarios of droplet impact on inclined surfaces (left). The leakage of the acoustic energy/pressure into the liquid medium is along the Rayleigh angle (*θ*_R_). *δ* is the distance between the impact and detachment points. *θ*_rebounc_ is the angle between the surface normal vector and the line connecting the separation point to the droplet tip at the separation moment in an anticlockwise direction. *f*_SAW_ is generated by the SAW. *L*_cl_ is the tangential diffusion distance of the droplet along the surface. *L*_f_ is the distance between the two tips of the droplet when the droplet is separated. And sequential snapshots of a water droplet impacting on the solid surface with an inclination angle of 15° (right). Reproduced with permission.^[^ ^78^ ^]^ Copyright 2020, American Chemical Society. c) Photos of droplet impact without SAW (left) and with SAW (right) on no treatment (NTS), a superhydrophobic (GTS), and SLIPS (SLIPs1) surface. Reproduced under terms of the CC‐BY license.^[^ ^79b^ ^]^ Copyright 2022, The Authors, published by American Chemical Society. d) Unified morphology map of the rebound and splashing when a droplet impacts the cold superhydrophobic surface under different *Web* numbers and surface temperatures. Reproduced with permission.^[^ ^72a^ ^]^ Copyright 2021, Elsevier B.V. e) Dynamics of droplet impacts on superhydrophobic surfaces: Stationary surface (top). rotating surface of *ω*_surf_ = 209 rad s^−1^ (middle). High‐speed rotating surface of *ω*_surf_ = 628 rad s^−1^ (bottom). Reproduced with permission.^[^ ^90^ ^]^ Copyright 2022, American Chemical Society.

There are differences in the impact angles between droplets and solids in the practical application environment. It is of great significance to understand the impact dynamics at different angles for the practical application of superhydrophobic materials. Increasing the tilt angle of the base material surface aids in reducing the fall rebound time, thereby enhancing the rebound effect. It can increase the critical transition *Web* number and help to resist puncture (**Figure** **5**a). The normal velocity component serves as the parameter that controls the piercing transition.^[^ ^82^, ^87^ ^]^ As the angle of inclination increases, the normal component of gravity decreases. As a result, the droplet has more kinetic energy during the retraction stage, which results in complete separation of the droplet from the surface.

Additionally, the elasticity of the surface affects the contact time of the droplet.^[^ ^81^, ^88^ ^]^ For elastic substrates, the effective acoustic impedance is lower than that of rigid substrates, reducing the water hammer pressure.^[^ ^89^ ^]^ In terms of energy, the elastic surface structure method relies on droplets as an energy storage mechanism (surface energy) during impact and recoil processes. The elasticity of the substrate increases so that the kinetic energy of the droplet is converted to surface energy and the elastic energy storage of the substrate, thereby replacing a single conversion to surface energy. Upon impact, the elasticity of the substrate provides an upward force that reduces the droplet impact time and increases the critical transition height.^[^ ^81^ ^]^

Various environmental conditions can effectively modify or postpone the C‐W transition. By applying a surface acoustic wave (SAW), the energy balance of the droplet can be changed from deposited to rebounding (Figure 5b).^[^ ^78^ ^]^ The combination of SAW and liquid injection on the porous surface also effectively changed the droplet impact dynamics, changing the droplet impact state from deposition to full rebound (Figure 5c).^[^ ^79b^ ^]^ On the supercooled surface, a decrease in surface temperature enhances the adhesion between the solid and liquid, leading to an increase in the contact time of the droplet and a reduction in the rebound height. As the surface temperature decreases further, the complete rebound behavior of the droplet changes to a partial rebound behavior, and the fixed droplet volume increases (Figure 5d).^[^ ^72a^ ^]^ In addition to the stationary surface, the contact time of impact droplets on a rotating superhydrophobic surface is reduced, thereby augmenting the resistance of puncture capability (Figure 5e).^[^ ^90^ ^]^

### 3.3 Evaporation

Evaporation is a prevalent physical occurrence in everyday life. Since wetting involves both solid and liquid phases, it is unavoidably influenced by evaporation. The decrease in volume and convection of heat complicate the interaction between the droplet and the substrate.^[^ ^91^ ^]^ Comprehending the evaporation kinetics of droplets in the C state and its mechanisms and influencing factors are of guiding significance for the design of a stable superhydrophobic surface.

**Figure 6:**
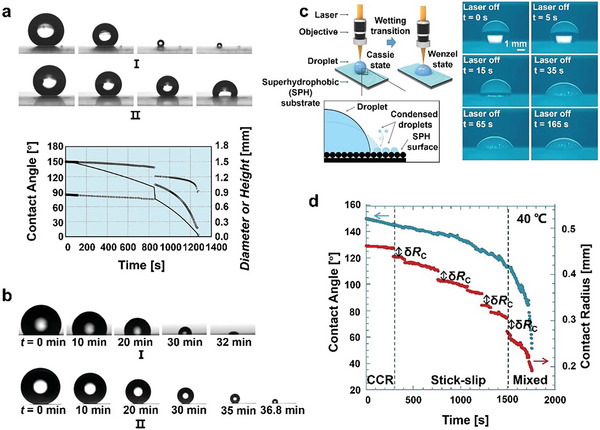
a) Images of droplet evaporation (top): I shows a droplet where the collapse from the C state occurs late in the evaporation. II shows a droplet where the collapse from the C state to the W state occurs at a middle stage during the evaporation and evolution with time of the CA (◊) and height (solid curve) and contact diameter (+) during the evaporation of a droplet of water where an obvious collapse occurs during the initial evaporation period (bottom). Reproduced with permission.^[^ ^92^ ^]^ Copyright 2005, American Chemical Society. b) Instantaneous images of an evaporating sessile droplet placed on (I) the smooth hydrophobic surface, and (II) the hierarchical superhydrophobic surface. Reproduced with permission.^[^ ^95^ ^]^ Copyright 2013, American Chemical Society. c) The schematic illustrations of the laser‐induced droplet evaporation (left) and the side views of the droplet evaporation during the laser heating (right). Reproduced with permission.^[^ ^97^ ^]^ Copyright 2018, Elsevier Ltd. d) Temporal evolution of the CA and the contact radius of a sessile droplet at 40 °C. The distinct evaporation configurations (CCR; moving contact line with stick–slip events; MM) are divided by vertical dotted lines. Reproduced with permission.^[^ ^99^ ^]^ Copyright 2014, Elsevier Inc.

McHale et al.^[^ ^92^ ^]^ first explored the evaporation behavior of superhydrophobic droplets on periodic microcolumn surfaces. It was found that the droplet would collapse during evaporation, which was the C‐W transition (**Figure** **6**a). The evaporation modes of droplets on solid surfaces can be divided into three types: constant contact angle mode (CCA) (the CA of droplets does not change or changes very little during the evaporation process, and the volume reduction is mainly determined by the movement of the contact line); constant contact radius mode (CCR) (the contact radius of droplets does not change or changes very little during evaporation, and the volume reduction is mainly determined by the reduction of CA); the mixing mode (MM) (the droplet volume decreases as both the CA and the contact radius decrease). The initial evaporation mode commences in either CCA mode or CCR mode. The evaporation process is carried out in an alternation of CCA and CCR modes and usually ends in MM mode.^[^ ^93^ ^]^ The difference in initial mode is explained by the difference in CAH. During the initial stage of evaporation, high CAH tends to be a CCR, and the CA decreases. Conversely, low CAH tends to be CCA and drop radius decreases.^[^ ^94^ ^]^ Dash et al.^[^ ^95^ ^]^ verified that the initial phase of evaporation tends to be in a CCA on hierarchical structure surfaces with low CAH, or even without a CCR throughout the evaporation process (Figure 6b). The occurrence of CCA indicates that droplets remain in the C state, and prolonging the CCA stage can effectively prolong the C state. Aldhaleai et al.^[^ ^96^ ^]^ verified that a CCA pattern emerges during the initial stage of evaporation with a small packing fraction and a low CAH (CAH ≤ 5°).

The investigation of the C‐W transition mechanism is of great significance to understand droplet evaporation behavior on a superhydrophobic surface. Li et al.^[^ ^93^ ^]^ explored the C‐W transition by discussing the driving force and resistance of TPCL. They determined that a C‐W transition will occur when the forward propelling force exceeds the resistance, and the contact line's longitudinal behavior plays a crucial role. The droplet's radius is a vital parameter, as a decrease in radius leads to the droplet's contraction and an increase in its internal pressure.^[^ ^56^ ^]^ When the radius is reduced to the critical value, the driving force of C‐W transition will be greater than the resistance. The equation for calculating the critical radius is as follows:^[^ ^93^ ^]^

(16) $$r_{C-W}=\frac{d}{2}\;\left(1-\frac{1}{f_{1}}\right)\frac{\sin\theta }{\sin\theta_{Y}-\left|\sin\theta_{\mathrm{adv}}\right|}$$

Where, *d* is diameter of pillars. In the presence of a local heat source, the evaporating droplet undergoes a rapid C‐W transition. The mechanism of the transition is due to the filling of steam condensation in the superhydrophobic microstructure (Figure 6c).^[^ ^97^ ^]^

According to the droplet evaporation theory, the effective diffusion area of a W state droplet in CCR mode is larger than that of C‐state droplet in CCA mode, due to a fixed contact area, and the droplet evaporation rate is the fastest. In addition, the high CA of C hindered the gas diffusion near the contact line, causing a slower diffusion speed.^[^ ^98^ ^]^ Ramos et al.^[^ ^99^ ^]^ verified that there is a linear relationship between the volume of CCR and the total evaporation time on the substrate heated to different temperatures, while CCA exhibits a power law relationship: $V^{\frac{2}{3}}\propto t$. On the structured solid surface, droplet evaporation exists in a stick‐slip mode. Due to the contact between the volume and the base in the process of reduction, which has energy fluctuations in thermodynamics, the contact line alternates in fixing and de‐pinning, showing a platform‐like discontinuous behavior.^[^ ^99^ ^]^ Stick‐slip behavior can also be observed in the evaporation behavior of large droplets, which is due to the increasing influence of gravity on the droplets (Figure 6d). The liquid meniscus sinks into the rough structure of the material surface due to gravity, amplifying the adhesion force. As the evaporation process reduces the droplet's size, it must overcome adhesion forces to transition to the next lower energy state.^[^ ^96^ ^]^

Droplet evaporation kinetics is affected by surface characteristics^[^ ^100^ ^]^ and environmental conditions (e.g., temperature^[^ ^96^ ^]^ and pressure^[^ ^101^ ^]^). Jung et al.^[^ ^102^ ^]^ designed superhydrophobic surfaces with different diameter, height, and spacing of microcolumns to explore the influence of microcolumn size on the stability of the C state during evaporation and found that samples with a large size and the same spacing factor had a larger critical transition droplet radius. This indicates that small‐scale surface is more conducive to the design of stable superhydrophobic surface in C state. Li et al.^[^ ^93^ ^]^ found that the smaller the microcolumn diameter, the more effectively it could prevent the C‐W transition. In terms of surface parameters, small size structures and hierarchical structures were more conducive to prolong the C state when droplets evaporated on the superhydrophobic surface. Additionally, when ambient pressure is different from atmospheric pressure, low pressure could facilitate the C‐W transition, triggered by the reduction of surface tension under low pressure conditions.^[^ ^101a^ ^]^

### 3.4 Vibration

When a droplet interacts with a surface, it can take the form of an impact that generates vibrations, for example, the blades shake when pesticides are sprayed and raindrops strike the vibrating wings. Vibration with certain amplitudes and frequencies usually affects the wetting and expanding behaviors of droplets. Bormashenko et al.^[^ ^103^ ^]^ have experimentally demonstrated that vibration can trigger the C‐W transition in water droplets. The investigation of the wetting transition and dynamic behavior of droplets on a vibrating surface is conducive to the development of a stable superhydrophobic surface in the C state, thus prolonging its service time. Vibrations are given in both horizontal^[^ ^104^ ^]^ and vertical^[^ ^105^ ^]^ directions. Droplet vibrations are usually driven by mechanical vibrations and SAW. Based on the frequency and amplitude of the vibration, two distinct types of droplet characteristic modes can be identified: At low amplitudes, the mode with a pin contact line is dominant; at higher amplitudes, the contact line oscillates at the excitation frequency and the contact line moves.^[^ ^106^ ^]^

**Figure 7:**
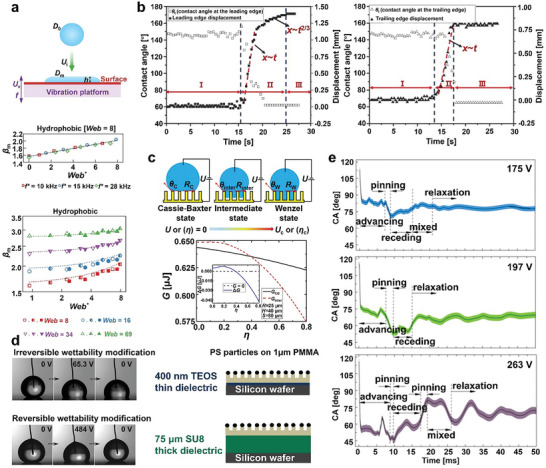
a) Characterization of droplet spreading on various surfaces. Schematic diagram of a droplet impacting on the vibratory surface, where, *D*_m_ and *h* represent the maximum diameter and the droplet's height at the maximum spreading, respectively (top). Plot of variation of the maximum diffusion factor and *Web* ^*^ at different frequencies on hydrophobic surfaces (middle). Plot of the variation of the maximum spreading factor *β*_m_ versus the *Web* ^*^ on hydrophobic surfaces. Open symbols, semi‐filled symbols, and solid symbols identify that f^*^ = 10, 15, and 28 kHz, respectively. Reproduced with permission.^[^ ^107^ ^]^ Copyright 2021, Elsevier Inc. b) Spreading dynamics and variation in the CA associated with the (left) leading (edge away from the interdigitated transducer) and (right) trailing (edge close to the interdigitated transducer) edges of the drop under SAW excitation. Reproduced under terms of the CC‐BY license.^[^ ^108^ ^]^ Copyright 2020, The Authors, Published by AIP Publishing. c) Different wetting states of a droplet on a microtextured surface in the presence of an external electric voltage: C state, intermediate composite state, and W state (top). Representative total interfacial free energies of the C and the intermediate states of a droplet on a micropillar‐patterned surface: *G*_CB_ and *G*_inter_, respectively, as a function of electrowetting number (bottom). Reproduced under terms of the CC‐BY license.^[^ ^52a^ ^]^ Copyright 2021, The Authors, Published by American Physical Society. d) Droplet snapshots showing an electrowetting experiment on a thin dielectric layer (400 nm TEOS) and a thick dielectric layer (75 µm SUB). Reproduced with permission.^[^ ^113^ ^]^ Copyright 2018, American Chemical Society. e) Instantaneous CA variation with time for a conducting water drop actuated at 175, 197, and 263 V. The shaded regions in the graphs shown represent the uncertainty. Reproduced with permission.^[^ ^117a^ ^]^ Copyright 2022, American Physical Society.

Throughout the transition process, the droplet experiences three states: In State 1, the CA remains constant while the interface oscillates symmetrically. In State 2, the droplet's CA decreases rapidly, the contact radius increases, and droplet diffusion occurs. Song et al.^[^ ^107^ ^]^ discovered that the diffusion diameter of the droplet is related to the vibration *Web* number ( $Web^{*}=\;\rho U_{V}^{2}D_{0}/\gamma$, where, *U*_v_ is the velocity amplitude of vibration) but not to the vibration frequency (**Figure** **7**a). The diffusion velocity is directly proportional to time (*x*  ≈  *t*). Subsequently, the diffusion velocity of the leading edge (the edge away from the SAW source) decreases, following a power‐law pattern with time (*x*  ≈  *t* ^2/3^). The contact line of the trailing edge (the edge close to the SAW source) remains stationary, halting the diffusion process. State 3: The contact line remains fixed and the droplet vibrates horizontally (Figure 7b). The total transition time is positively correlated with droplet volume. The time of stage 1 is most affected by volume, and the time increases with the increase in volume.^[^ ^108^ ^]^ Throughout the entire process of SAW activity, capillary waves emerge at the interface of droplets. These capillary waves alter the shape of the interface, prompting fluid movement and adjustments in the pressure distribution at the interface.^[^ ^107^, ^109^ ^]^ Concurrently, the viscous effects within the droplet and the dissipation of the TPCL are impeded.^[^ ^110^ ^]^

It is crucial to grasp the mechanism and critical conditions of the C‐W transition during droplet vibration. When the external vibration of a certain energy is applied to the entire wetting system, the pressure jump generated by the droplet vibration pushes the droplet into the surface groove, causing the C‐W transition.^[^ ^103a^ ^]^ The pressure caused by vibration mainly acts on the TPCL, and the C‐W transition occurs when the constant force per unit length exceeds a certain threshold.^[^ ^103b^ ^]^ The dynamic pressure value, which is positively correlated with the Web^*^ value, promotes droplet expansion behavior.^[^ ^107^ ^]^ The transition occurs when the input energy is above the threshold. For the vibration energy threshold provided by SAW, its size is:^[^ ^108^ ^]^

(17) $$E\approx \frac{r^{*}}{x}\pi R_{c}^{2}\gamma_{\mathrm{SL}}-\gamma_{\mathrm{SG}}$$

Where, *r* ^*^, *x*, and *R*_c_ are the average surface roughness, particle radius, and contact line radius of the droplet, respectively.

### 3.5 Electrowetting

In the presence of an additional electric field, the droplet will also undergo a C‐W transition. A wettability transition occurs only when the energy input from the electric field overcomes the energy barrier of the wettability transition. The maximum free energy of the system always occurs after the droplet touches the bottom of the groove.^[^ ^12^ ^]^ The energy of the intermediate transition state is significantly reduced in the presence of the electric field, resulting in a lower energy than the C state. As the applied voltage elevates, the energy barrier of droplets penetrating into the rough substrate gradually decreases until a critical voltage is reached, and the energy barrier decreases to zero when droplets penetrate into the rough substrate. As the voltage continues to escalate further, the infiltration of the droplets occurs spontaneously until a wetting transition occurs when the droplets make contact with the substrate (Figure 7c).^[^ ^52a^ ^]^ Manukyan et al.^[^ ^111^ ^]^ discovered that the stability of the C state under electrowetting is determined by the balance between Maxwell stress (π  =  εε_0_ *E* ^2^/2, where, *ε*_0_ is the vacuum dielectric constant and *ε* is the relative dielectric constant of the C state cavity intermediates, i.e., *ε* = 1 for air) and Laplace stress (Δ *p*_L_ =  σκ, where, *κ* is the curvature of the water–air interface). The critical voltage is affected by surface parameters and droplet volume. An increase in the transition energy barrier resulting from changes in surface parameters leads to an increase in the critical voltage. Similarly, an increase in volume also results in an increase in the critical voltage.^[^ ^52a^ ^]^ The superhydrophobic surface of nanoscale structures effectively enhances the stability of the C surface under an electric field, and even realizes the reversible cycle between the C and W states.^[^ ^112^ ^]^ Moreover, the thickness of the dielectric layer influences the critical voltage by modulating the electrostatic range. In comparison to a thick electrostatic layer, the electrostatic force near the contact line of a thin dielectric layer intensifies, leading to an increased local mean curvature of the droplet. As the dielectric layer thickness augments, the system becomes more capable of tolerating an elevated critical voltage (Figure 7d).^[^ ^113^ ^]^ Similarly, reversible wetting can occur when the dielectric layer thickness reaches a specific threshold.^[^ ^114^ ^]^ Any non‐wetting liquid–solid interface, where the solid surface has a surface charge density and the liquid has dissolved ions and a high dielectric constant, can lead to charged droplets. The charge depends on the liquid–solid interface area.^[^ ^115^ ^]^ Under the influence of an electric field, the uneven charge distribution causes the droplet to penetrate into the shape of a cone, and the electrowetting effect near the sidewall becomes more complex.^[^ ^116^ ^]^

The behavior of the C‐W transition under an electric field is closely related to electrowetting saturation, CA instability, and stick‐slip motion.^[^ ^116^ ^]^ The CA of the droplet decreases with the increase in voltage until it reaches the braking voltage (i.e., CA saturation) and remains fixed.^[^ ^117^ ^]^ Saturation effects can be caused by charge accumulation in the liquid and dielectric layers, limited resistance, current flow in the material, and instability of the contact line.^[^ ^116^ ^]^ The application of an electric field brings certain vibrations to the droplet and generates interfacial capillary waves, which cause unstable fluctuations of the CA value. However, this fluctuation will weaken with the viscous effect of the droplet over time (Figure 7e).^[^ ^117a^ ^]^

## 4 Structural Factors Affecting the Stability of C State

### 4.1 Intrinsic Hydrophobic

The intrinsic hydrophilic and hydrophobic abilities of materials are important factors affecting the wettability of materials. Initially, only substances with low surface energy can be used to create superhydrophobic surfaces. Until a later stage, it was also possible to construct superhydrophobic materials using hydrophilic substances by designing unique surface structures, reflected in CA values greater than 150°.^[^ ^118^ ^]^ Nonetheless, for the stabilization time of the C state, the superhydrophobic surfaces constructed by intrinsically hydrophobic substances are maintained longer than those constructed by intrinsically hydrophilic substances, even if the apparent CA is the same. In other words, intrinsically hydrophobic substances are more conducive to the improvement of C state stability. As the hydrophobic rough structure exhibits weak intermolecular gravitational forces at the liquid–solid surface, a larger driving force is required to make the water film invade the groove, resulting in a higher breakthrough barrier. The C state is more stable at the grooved surface because a higher energy barrier needs to be overcome to trigger the C‐W transition. In contrast, the W state is more favorable on surfaces with hydrophilic grooves and is therefore more likely to trigger the C‐W transition.^[^ ^12^ ^]^

From the point of view of the C‐W transition mode, when the droplet is subjected to a certain force, the subsurface interface sags, and the critical state of the contact line breaking through the pin is the moment when the subsurface CA exceeds the *θ*_adv_. A high *θ*_adv_ value can effectively increase the breakthrough energy barrier. The intrinsic hydrophobic substance can improve the *θ*_adv_ effectively.^[^ ^66^ ^]^

### 4.2 Structured Hydrophobic

The droplet and the surface of the target material finally reach an equilibrium state through various interactions, which may be a C state, a partial‐C state, a W state, and other states. The factors affecting this final state can be summarized as follows:

1. Physical properties of the droplet (surface tension, dynamic viscosity, volume, density, etc.);
2. External force (pressure size, impact velocity, temperature, vibration frequency, etc.);
3. The surface parameters of the target material (structural size, structural shape, and structural level) and intrinsic CA.

This section deals only with water (density, *ρ* = 997 kg m^−3^; surface tension, *σ* = 72.8 mN m^−1^; and dynamic viscosity, *µ* = 0.89 mPa s^−1^ at laboratory room temperature) on the surface of the material wetting state and transition.

#### 4.2.1 Structure Size

##### Height

**Figure 8:**
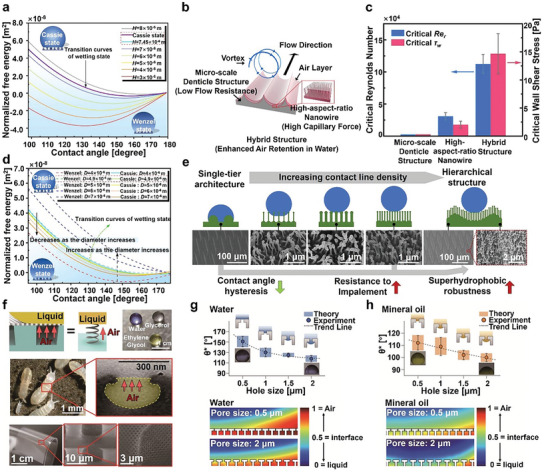
a) Variations of normalized free energy as a function of apparent CA on the irregular micro‐structured surface under the wetting state for different protrusion heights. Reproduced with permission.^[^ ^44a^ ^]^ Copyright 2022, Elsevier B.V. b) Schematics of air layer holding enhancement of water‐repellent hybrid structure. c) Critical Reynolds number (blue) and critical wall shear stress (red) of different surface structures under water dynamic flow conditions. Reproduced under terms of the CC‐BY license.^[^ ^120^ ^]^ Copyright 2018, The Authors, Published by Springer Nature. d) Variations of normalized free energy as a function of apparent CA on the surface of irregular micro‐structured surface for different protrusion maximum diameters. Reproduced with permission.^[^ ^44a^ ^]^ Copyright 2022, Elsevier B.V. e) Schematic illustration of improving surface superhydrophobicity and puncture resistance by increasing line contact density. Reproduced with permission.^[^ ^122^ ^]^ Copyright 2022, Elsevier Ltd. f) Repulsive air‐spring based omniphobic surface that mimicked skin of springtail. g) Changes of the CA (top) and simulation of air‐trapping (bottom) of water by the nanohole size. h) Changes of the CA (top) and simulation of air‐trapping (bottom) of mineral oil by the nanohole size. Reproduced with permission.^[^ ^123^ ^]^ Copyright 2019, American Chemical Society.

The height of the microcolumn/microgroove is an important parameter of material surface morphology. Researchers found that the height of the microcolumn/microgroove can affect the wettability of the material surface. The effect of microcolumn/microgroove height on the wetting transition occurring on superhydrophobic surfaces has been examined through experimental and thermodynamic analyses. The findings of these studies indicate that the effect of microcolumn/microgroove height on the W state is more significant than its impact on the C state. Under the influence of the contact range due to the air pocket in the microcolumn/microgroove, the contact range of the C state is mainly situated at the column's apex, and its free energy is not affected by the height or is very slightly affected. In the W state, the droplet completely touches the solid surface. The height affects the solid–liquid contact area, and increasing the height leads to an increase in the free energy of the W state (**Figure** **8**a).^[^ ^44a^ ^]^ The decrease in the free energy difference between C state and W state is not conducive to the transition of the system to W state and is conducive to the stability of C state. Gao et al.^[^ ^119^ ^]^ simulated the behavior of height affecting the wettability state of droplets. When the height of the microcolumn was increased, the wetting state of droplets on the surface transitioned from the W state to the C state, indicating that a high microcolumn was conducive to the C state. Jo et al.^[^ ^120^ ^]^ constructed nanowire structures with high aspect ratios to enhance capillary forces, maintain air cavitation, improve air holding capacity at the water surface underwater, and prolong the C state (Figure 8b,c).

##### Microcolumn Diameter/Microcolumn Spacing

In terms of the contact area fraction between the droplet and the material surface, the diameter and spacing of the microcolumn are important influencing parameters. The researchers examined the effects of these two parameters on wettability separately by maintaining control over variables. In this particular section, the influence of microcolumn diameter (microcolumn spacing) on the wettability of the material surface is discussed using a fixed area fraction. That is, for a fixed area fraction, the microcolumn diameter is competitively related to the microcolumn spacing. Increasing the microcolumn diameter leads to an increase in the energy of the W state and a decrease in the energy of the C state. In terms of the energy of the two states, the increase in diameter favors the stability of the C state (Figure 8d).^[^ ^44a^ ^]^ Correspondingly, an increase in diameter corresponds to a decrease in spacing. The energy barrier of the C‐W transition increases as the spacing decreases.^[^ ^66^, ^121^ ^]^ Zhang et al.^[^ ^122^ ^]^ enhanced the stability of C states on superhydrophobic surfaces by increasing the density of microcolumns (Figure 8e).

For the microporous surface, the reduction in hole diameter makes it challenging for droplets to invade, which is more conducive to the stability of the C state.^[^ ^52a^ ^]^ Seo et al.^[^ ^123^ ^]^ created an omniphobicity surface by mimicking the surface structure of springtails and found that the narrowing of the hole diameter gap could enhance air retention and further improve omniphobicity (Figure 8f–h). For the cavity structure, reducing the cavity diameter raises the energy barrier for breakthrough, allowing it to endure higher pressure and ensuring a more stable C state.^[^ ^124^ ^]^

#### 4.2.2 Structural Shape

According to thermodynamic analysis, the energy barrier for droplet wetting varies depending on the micromorphology. The influence of specific micromorphology on the wetting transition can significantly contribute to the establishment of a stable C state. According to the existing experimental conditions, most preparation methods lack the precision to regulate structure size, yet the construction of the general morphology can still meet certain requirements. Wan et al.^[^ ^125^ ^]^ successfully grew α‐FeOOH nanoneedles and nanoflowers on carbon aerogel by chemical precipitation. Rajesh et al.^[^ ^126^ ^]^ successfully grew ZnO nanocrystalline/micron rod arrays on a metal substrate by hydrothermal method. Chen et al.^[^ ^127^ ^]^ loaded active reactive substances into microspheres and constructed microsphere protrusions on the substrate through heat‐induced polymerization. The surface morphology of materials can be accurately controlled by lithography and the template method, albeit at a high cost.^[^ ^128^ ^]^ The innovation and simplification of the preparation methods of the material morphology can effectively enhance the structural design to achieve a stable C state. In terms of the structural designs that have been studied, they are mainly divided into simple structures and reentrant structures. This paragraph will analyze the impact on the C state from both these perspectives.

##### Simple Structures

According to the shape system, the simple microstructure of the material surface can be categorized into three types: upper wide and lower narrow type, rectangular type, and upper narrow and lower wide type. Additionally, according to the sidewall internal angle value, it can be further subdivided into an inverted trapezoid, square, parabola, trapezoid and triangle, with the value increasing accordingly. As for the wetting transition energy barrier, the height of the microstructure, the diameter of the upper surface, and the spacing between the microcolumns all affect the transition energy barrier value, which is reflected in the stability of the C state. The analysis shows that for the same parameter, the structure with a wide top and narrow bottom has the largest transition energy barrier, which means that the C state is the most stable. Narrow at the top and wide at the bottom are the least stable. Rectangular structures fall somewhere in between. Reducing the sidewall internal angle can enlarge the range of apparent CA, resulting in an increased energy barrier.^[^ ^129^ ^]^

The effect of the hole type/groove type of construction is the same as that of the column shape. If the hole/groove solid wall shape is an inverted trapezoid, the C state becomes more stable and can withstand the maximum hydraulic pressure.^[^ ^38^, ^130^ ^]^ The CA of the microgroove along the oblique wall direction varies with the angle of the microcavity along the longitudinal direction resulting in a change in curvature when the droplet is immersed. Droplet shapes with different curvatures imply a Laplace pressure gradient that causes the droplet to move in a direction favorable to the gradient. As a result, the sliding free energy gradient of the droplet changes.^[^ ^46^ ^]^ The hole/groove inner angle is an important parameter. According to the inner angle wetting equation of liquid under microgravity (α + θ < π/2, where, *α* is the inner half angle), capillary forces drive the liquid to flow along the inside corners.^[^ ^131^ ^]^ Xiang et al.^[^ ^42^ ^]^ established the expansion equation for gas:

(18) $$\alpha +\left(\pi -\theta \right)<\frac{\pi }{2}$$

**Figure 9:**
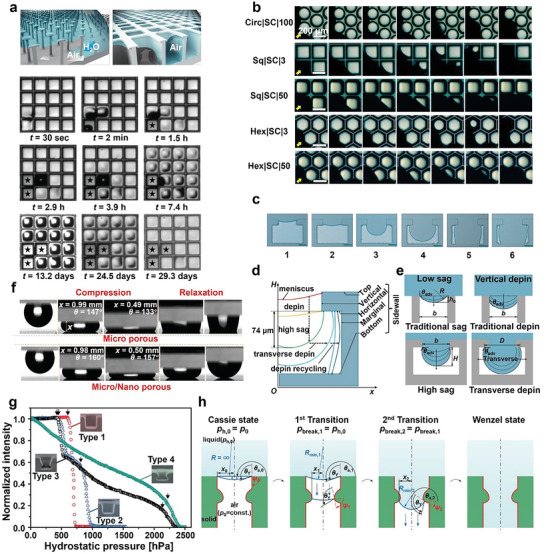
a) Isometric schematics of liquid on an array of doubly reentrant pillars and square‐shaped doubly reentrant cavities (top). Optical micrographs (top view) of an array of square‐shaped doubly reentrant cavities on a silica surface immersed underwater. Cavities filled with water are marked with a star (★) (bottom). Reproduced with permission.^[^ ^132^ ^]^ Copyright 2017, American Chemical Society. b) Silica surfaces with simple cavities (SCs) immersed in hexadecane. Yellow arrows indicate the direction of the advancing liquid at the onset of immersion. Reproduced under terms of the CC‐BY license.^[^ ^124^ ^]^ Copyright 2018, The Authors, Published by Springer Nature. c) The graphs of critical meniscus morphology of wetting transition in reentrant microstructures. Scale bar: 100 µm. d) Diagram of changes in critical meniscus during wetting transition. e) Comparison diagram of infiltrated meniscus changes between simple microcolumn and reentrant microcolumn. Reproduced with permission.^[^ ^135^ ^]^ Copyright 2017, American Chemical Society. f) Comparative photos of compression and release tests using a micro porous hydrophobic surface and a micro/nanoporous surface. Reproduced with permission.^[^ ^139^ ^]^ Copyright 2014, American Chemical Society. g) Impact of the shape of sidewall profiles on the wetting transition barrier. The decrease of the normalized intensity of the scattered light from 1 to 0 represents C‐W transition. h) Diagram of pressure‐induced wettability transition in a side wall containing a raised structure. Reproduced with permission.^[^ ^121^ ^]^ Copyright 2022, American Chemical Society.

Small sidewall inner angle can cause gas expansion effect. The surface's resistance to gas infiltration has improved. Even when the surface is completely infiltrated, it can be covered with a gas film by injecting gas to preserve the C state. In contrast to columnar structures, independently spaced cavity structures prevent catastrophic wetting transitions in the presence of local structural damage/defects or immersion in wetting fluids (**Figure** **9**a).^[^ ^132^ ^]^ Cavity structures come in a variety of shapes, including triangles, squares, hexagons, and circles. Of these, cavities with sharp corners begin to absorb liquid earlier than those with rounded corners. The fillet cavity structure can maintain a longer C state under hydraulic pressure (Figure 9b).^[^ ^124^ ^]^

##### Reentrant Structure

Reentrant structures can be used to construct superhydrophobic surfaces because of their unique folding topology.^[^ ^123^, ^133^ ^]^ In addition, reentrant structures also play a unique advantage in constructing long‐lasting superhydrophobic surfaces. The reentrant structure has been observed on the surface of springtails (*Collembola*), allowing them to breathe in rain‐flooded environments.^[^ ^134^ ^]^ It has been discovered that the T‐shaped or double‐reentrant microcolumn structure possesses a higher critical wettability transition pressure than the straight column structure. Reentrant structures can resist high pressures on surfaces with low intrinsic CA, whereas straight column types require intrinsically hydrophobic surfaces.^[^ ^134^ ^]^ He et al.^[^ ^135^ ^]^ explored the wettability transition mechanism of reentrant structures and confirmed that high sagging and transverse depinning are the key factors affecting the wettability transition. The interfacial tension of the large curvature interface formed by the high sagging is used to resist the external pressure (Figure 9c–e). And put forward the design criteria for the reentrant structure:

(19) $$c>\frac{b}{\sin\theta_{\mathrm{adv}}}$$

(20) $$h>\frac{b\left(1-\sin\theta_{\mathrm{adv}}\right)}{2\sin\theta_{\mathrm{adv}}}$$

Where, *b*, *c*, and *h* are the parameters of reentrant microstructures. Stable C state surface can be prepared by reasonable parameter design.

#### 4.2.3 Structure Level

The researchers were inspired by natural surfaces like lotus leaves and found that on these surfaces, the rough structures are micro–nano composite structures (i.e., hierarchies structures) that exhibit both high CA values and stable C states.^[^ ^136^ ^]^ Compared with the microcolumn structure, the equilibrium CA value of the surface of the hierarchical structure increases, representing the most stable state of the system's free energy. The value is close to the low position of C state capacity.^[^ ^137^ ^]^ By means of computational fluid dynamics simulation, nanostructures were introduced into the top of the microcolumn. The critical height and critical distance of the Wenzel‐Cassie wetting state transition (reversal of the C‐W transition) are lower and wider, respectively, compared with the single microcolumn, indicating that the micro–nano composite structure is favorable for the droplet to be located in the C state. Moreover, the wide spacing of nanostructures is conducive to the C state.^[^ ^119^, ^138^ ^]^ Tuvshindorj et al.^[^ ^139^ ^]^ compared the compressive stability of single‐scale and double‐scale micro–nano porous surfaces by squeezing experiments and proved that the double‐scale micro–nano structure has a higher critical pressure for transition and a more stable C state (Figure 9f). Pan et al.^[^ ^140^ ^]^ made a comparison by constructing a multi‐scale structure and concluded that an increase in hierarchical level and structural complexity can effectively increase the critical pressure that the surface can withstand. Because hierarchical structures can provide greater capillary force, they can increase resistance to water intrusion compared to microns or nanostructures.^[^ ^55^ ^]^ Teisala et al.^[^ ^141^ ^]^ not only achieved superamphiphobic surface behavior but also enhanced the stability of the air cushion by constructing hierarchical nanostructures. The micro‐nanocomposite structure is divided into the nanostructure at the top of the microcolumn and the sidewall to explore the main influencing parameters, that is, the difference between smooth sidewall and rough sidewall. The hierarchical structure of the top of the microcolumn can effectively increase the contact gas fraction and improve the CA.^[^ ^142^ ^]^ At the same time, the ability of liquid intrusion between microcolumns is enhanced, that is, the intrusion energy barrier is reduced.^[^ ^143^ ^]^ The rough sidewall can effectively increase the apparent CA and the energy barrier of the wetting transition, which is conducive to the stability of the C state.^[^ ^144^ ^]^

The sidewall is the most important solid–liquid contact surface during the C‐W transition. The wettability transition kinetics and energy barrier are affected by the sidewall structure. Nosonovsky^[^ ^145^ ^]^ examined the influence of the microcolumn sidewall on wettability more than 10 years ago and found that the convex structure is conducive to the stability of the hydrophobic surface and increases the energy barrier of liquid moving down. Hensel et al.^[^ ^121^ ^]^ also confirmed that the presence of lateral wall bulges can increase the hydrostatic pressure of the transition (Figure 9g,h). An important step in the process of wetting transition is the slip occurring on the sidewall, which is determined by the *θ*_adv_ of the droplet on the sidewall. Fang et al.^[^ ^69b^ ^]^ found that the rough sidewall can effectively increase the *θ*_adv_. Compared with the smooth microcolumn, the CA of the sidewall of the smooth top microcolumn with the nanostructure sidewall increases, and the critical microcolumn spacing of the wetting transition on the surfaces of the fixed gas fraction increases. The structured sidewall helps to strengthen the pins of the TPCL on the sidewall of the cavity.^[^ ^36b^ ^]^ That is to say, the smooth top microcolumn with the same spacing, the microcolumn with the nano‐side wall liquid intrusion becomes difficult, and the C state is more stable.^[^ ^144^ ^]^

## 5 Conclusion

Superhydrophobic materials have been developed for various applications due to their low interaction force with water, making the water droplet present special wettability (water resistance) on surfaces. The existence of an air layer beneath the liquid reduces the contact area between the liquid and the solid, that is, the C state effectively reduces the solid‐liquid interaction force. For the W state, the liquid and the solid are completely contacted, resulting in a large increase in force and a decrease in superhydrophobicity. The C‐W transition is difficult to avoid in terms of thermodynamics because the C state is in high energy state and the W state is in low energy state. The transition energy barrier between the two states can be crossed under certain conditions. In this review, the dynamic behavior and mechanism of C‐W transition of water droplets under different conditions, including pressure, impact, evaporation, vibration, and the electrowetting environment, are described in detail. The external environment leads to an increase in system energy and the C‐W transition occurs. From the mechanical point of view, the driving force caused by the external influence makes the under‐liquid contact line break through the equilibrium position, which leads to the under‐liquid interface touching the substrate or the contact line sliding down and then contacting the substrate, that is, the contact transition or the depinning transition occurs. Increasing the wetting resistance can effectively alleviate the C‐W transition and prolong the usage life of the superhydrophobic surfaces.

The structure design of solid surface morphology has been proved to effectively improve the stability of C state. Effective structural design can greatly extend the critical pressure at which the transition of the superhydrophobic surface occurs, resulting in prolonged service life (e.g., the *Salvinia* leaf can maintain a long plastron layer underwater due to its special egg‐beater structure). In this review, the influence of structure on the C state is systematically analyzed in terms of structure size, structure shape, and structure level. It is concluded that a high and narrow structure is superior to a low and wide structure. The reentrant structure inspired by the springtails can effectively prolong the C state due to its advancement of high sagging and transverse depinning. The hole and cavity structures because, of their independence of the structural units prevent the continuous wetting of the liquid and prolong the service life. The elevation of the transition energy barrier caused by a multi‐scale hierarchical structure can effectively increase the immersion pressure, and the improvement of sidewall roughness is the key factor. In general, there are three main ways to extend the C state from the thermodynamic analysis of the structure:

1. Reduce the energy of the system in the C state;
2. Increase the energy of the system in the W state;
3. Increase the energy barrier of C‐W transition.

The existing structural design to extend the service life of the C state is limited and can cope with only relatively mild environmental conditions. While raising the external pressure, the duration of the C state will be shortened. C state is also very difficult to maintain under harsh conditions (e.g., an ultra‐high water pressure submarine operating environment). The diffusion of air into the liquid makes the C state difficult to maintain for a long time. At the same time, complex structure design is faced with the problems of difficult preparation and low wear resistance, which is also an important reason for the low commercialization of superhydrophobic surfaces. It is still necessary to explore and create structural designs and methods that can obtain the C state for a long time in the face of complex and demanding use conditions. At present, the active strategy of air injection and the passive strategy of electrolysis have been used to regenerate the air layer and extend the service life of the C state. But it also faces the disadvantages of inconvenient application and high energy consumption.

In short, how to obtain a long‐term C state surface in a simple and effective way is the key and difficult point for the practical application of superhydrophobic materials. Effective structural design is a promising and proactive strategy for future fabrication. Simple and low‐consumption preparation technology, high critical immersion pressure, long C state application time, and morphology structure retention ability are the directions that need to be explored and innovated in the future.

## Conflict of Interest

The authors declare no conflict of interest.
